# Invariant structural and functional brain regions associated with tinnitus: A meta-analysis

**DOI:** 10.1371/journal.pone.0276140

**Published:** 2022-10-18

**Authors:** John C. Moring, Fatima T. Husain, Jodie Gray, Crystal Franklin, Alan L. Peterson, Patricia A. Resick, Amy Garrett, Carlos Esquivel, Peter T. Fox

**Affiliations:** 1 Department of Psychiatry and Behavioral Sciences, University of Texas Health Science Center at San Antonio, San Antonio, Texas, United States of America; 2 Department of Speech and Hearing Science and the Beckman Institute for Advanced Science and Technology, University of Illinois at Urbana-Champaign, Champaign, Illinois, United States of America; 3 Research and Development Service, South Texas Veterans Health Care System, San Antonio, Texas, United States of America; 4 University of Texas at San Antonio, San Antonio, Texas, United States of America; 5 Department of Psychiatry and Behavioral Sciences, Duke University Medical Center, Durham, North Carolina, United States of America; 6 Hearing Center of Excellence, Wilford Hall Ambulatory Surgical Center, San Antonio, Texas, United States of America; Universidad de Chile, CHILE

## Abstract

Tinnitus is a common, functionally disabling condition of often unknown etiology. Neuroimaging research to better understand tinnitus is emerging but remains limited in scope. Voxel-based physiology (VBP) studies detect tinnitus-associated pathophysiology by group-wise contrast (tinnitus vs controls) of resting-state indices of hemodynamics, metabolism, and neurovascular coupling. Voxel-based morphometry (VBM) detects tinnitus-associated neurodegeneration by group-wise contrast of structural MRI. Both VBP and VBM studies routinely report results as atlas-referenced coordinates, suitable for coordinate-based meta-analysis (CBMA). Here, 17 resting-state VBP and 8 VBM reports of tinnitus-associated regional alterations were meta-analyzed using activation likelihood estimation (ALE). Acknowledging the need for data-driven insights, ALEs were performed at two levels of statistical rigor: corrected for multiple comparisons and uncorrected. The corrected ALE applied cluster-level inference thresholding by intensity (z-score > 1.96; *p* < 0.05) followed by family-wise error correction for multiple comparisons (*p* < .05, 1000 permutations) and fail-safe correction for missing data. The corrected analysis identified one significant cluster comprising five foci in the posterior cingulate gyrus and precuneus, that is, not within the primary or secondary auditory cortices. The uncorrected ALE identified additional regions within auditory and cognitive processing networks. Taken together, tinnitus is likely a dysfunction of regions spanning multiple canonical networks that may serve to increase individuals’ interoceptive awareness of the tinnitus sound, decrease capacity to switch cognitive sets, and prevent behavioral and cognitive attention to other stimuli. It is noteworthy that the most robust tinnitus-related abnormalities are not in the auditory system, contradicting collective findings of task-activation literature in tinnitus.

## Introduction

Subjective tinnitus is the perception of sound in the absence of any external sound source (i.e., an illusory percept). Tinnitus is commonly described as ringing, buzzing, whooshing, or a combination of sounds in one or both ears [1,2]. Tinnitus prevalence varies from 3%-14% in the general population [3]. Roughly 1–3% of individuals with tinnitus report significant functional impairment [4,5], including difficulties with sleeping, concentration, and communication. Combinations of tinnitus sounds (e.g., ringing *and* buzzing or buzzing *and* whooshing) cause significantly more impairment than a single percept [6]. Although tinnitus is a common, functionally disabling condition that has been described in the medical literature for millenia [7,8], the neurobiology of tinnitus remains unsolved. Over roughly the past two decades, a modest body of neuroimaging studies has emerged seeking to address this shortcoming.

Neuroimaging studies fall into two broad classes: functional and structural. In both classes, atlas-referenced coordinates are widely used, making the literatures amenable to coordinate-based meta-analysis (CBMA; [9–11]). Functional studies can be further subdivided into task-activation (TA) and resting-state (RS). The early neuroimaging literature in most disorders, including tinnitus, relied heavily on TA methodology. A distinct advantage of the task-activation approach is the high signal-to-noise ratio of the task-induced activations and, secondarily, of the superimposed inter-group, condition-related differences in activation. The TA literature was, from the outset, reliant on coordinate-based reporting. The cardinal limitations of the TA approach are: (1) regions probed are largely limited to those engaged by the task; (2) sensitivity is reliant on participant performance, which can be highly variable in clinical populations; and (3) there are a wide range of control tasks that are employed. The majority of the tinnitus TA literature has used sound stimuli as a probe, and this was meta-analyzed by Song and colleagues [12]. Tinnitus-related increases in activation were observed but, predictably, these increases were almost entirely limited to primary and secondary auditory cortices.

Between-group contrasts (disease vs control) of resting-state physiology using PET and SPECT have been reported in tinnitus since the late 1990s [13,14], prior to the widespread adoption of statistical parametric mapping using standardized coordinates for non-task studies in clinical cohorts. Reviews of this early literature were conducted by Adjamian [15] and Lanting [16]. More recently, resting-state fMRI (rs-fMRI) measures have been ascendant, exploiting both hemodynamic measures (e.g., arterial spin labeling; ASL) and BOLD-derived metrics of neurovascular coupling, most notably regional homogeneity (ReHo; [17]) and fractional amplitude of low-frequency fluctuations (fALFF; [18]). Here we refer to resting-state fMRI, PET, FDR-PET, and SPECT studies which apply mass-univariate statistics (the same operation is performed on each image voxel) and report in standardized coordinates as voxel-based physiology (VBP) studies. By reporting standardized coordinates, the VBP literature provides suitable input for CBMA. By assessing participants in the resting-state, this literature provides a regionally unbiased examination of the gray-matter physiology in persons with tinnitus, which is a distinct paradigm shift from the task-activation literature. Resting-state VBP studies using fMRI (60%; 15/25) form the bulk of the literature meta-analyzed here.

Resting-state contrasts of brain structure, also task independent, most often are performed using voxel-based morphometry (VBM; [19]) applied to T1-weighted MRI. VBM, like VBP, is a mass-univariate method in which spatially standardized images are contrasted group-wise to detect abnormalities too subtle to be recognized by visual inspection, but which are sufficiently reliable in location to be detected by group-wise contrasts. Like VBP studies, VBMs are regionally unbiased with the caveat that VBM—like VBP—are most sensitive for detecting gray-matter effects. VBM gray-matter studies make up the remainder (32%; 8/25) of the literature examined herein.

Activation/anatomical likelihood estimation (ALE: [9,20,21]) is the most widely utilized CBMA algorithm [22]. Although ALE CBMA was originally designed for task-activation meta-analysis and has been most extensively used for single-modality, (see BrainMap.org/pubs), this is not an intrinsic limitation of the ALE method. Rather, ALE assesses the spatial proximity of reported coordinates against a null hypothesis of a random distribution of the same volume and quality of data. ALE computes the convergence of findings based solely on location and is blind to magnitude and direction [23]. Therefore, this modality agnosticism allows ALE the flexibility to integrate findings across imaging methods [24,25], if to do so is logically appropriate. Co-localization of structural and functional alterations are found in numerous neurodegenerative and psychiatric disorders [25]. Since studies have found both structural and functional alterations related to tinnitus, it is appropriate to implement the ALE algorithm. Results will identify the disease effects related to gray matter alterations and the co-localization of both increases and decreases in resting-state function. In the present study, we combined resting-state VBP studies and VBM studies contrasting persons with tinnitus to healthy controls for a comprehensive assessment of tinnitus-related gray-matter alterations. Significant group contrasts, between tinnitus groups and control groups from each study were included in the meta-analysis, independent of whether results indicated increased/decreased GM/resting-state function. According to Müller et al. [26], multiple experiments with the same set of participants can compromise the validity of resullts. Therefore, the procedures of this meta-analysis included only one experiment per subject group.

Acknowledging the limited volume of the quantitative, coordinate-reporting literature in tinnitus and the necessity for data-driven etiological insights, CBMAs were performed at two levels of statistical rigor: confirmatory and exploratory. The more conservative approach applied two statistical thresholds: (1) intensity (*p* < 0.05) at the voxel level; and, (2) a correction for multiple comparisons at the cluster-forming level using family-wise error rate (FWE; 1,000 permutations, *p* < 0.05). Additionally, the fail-safe correction for missing data (publication bias) was applied [22,25]. The less conservative approach applied only voxel-wise thresholds, with no corrections for multiple comparisons or missing data. The intent of this exploratory analysis was to probe the available data as deeply as possible and thereby simulate hypothesis generation as well as to identify candidate nodes for network analyses.

The overall goal of the present study was to identify brain regions exhibiting tinnitus-related functional and structural alterations in the absence of task performance by applying CBMA to the VBP and VBP literatures. The null hypothesis of ALE CBMA is spatial non-convergence (i.e. a random data distribution). The hypothesis of the investigators was that this task-free approach would demonstrate abnormalities outside the confines of the auditory system and provide new, data-driven insights into the pathophysiology of tinnitus [27–29].

## Methods

### Literature search

**Fig 1:**
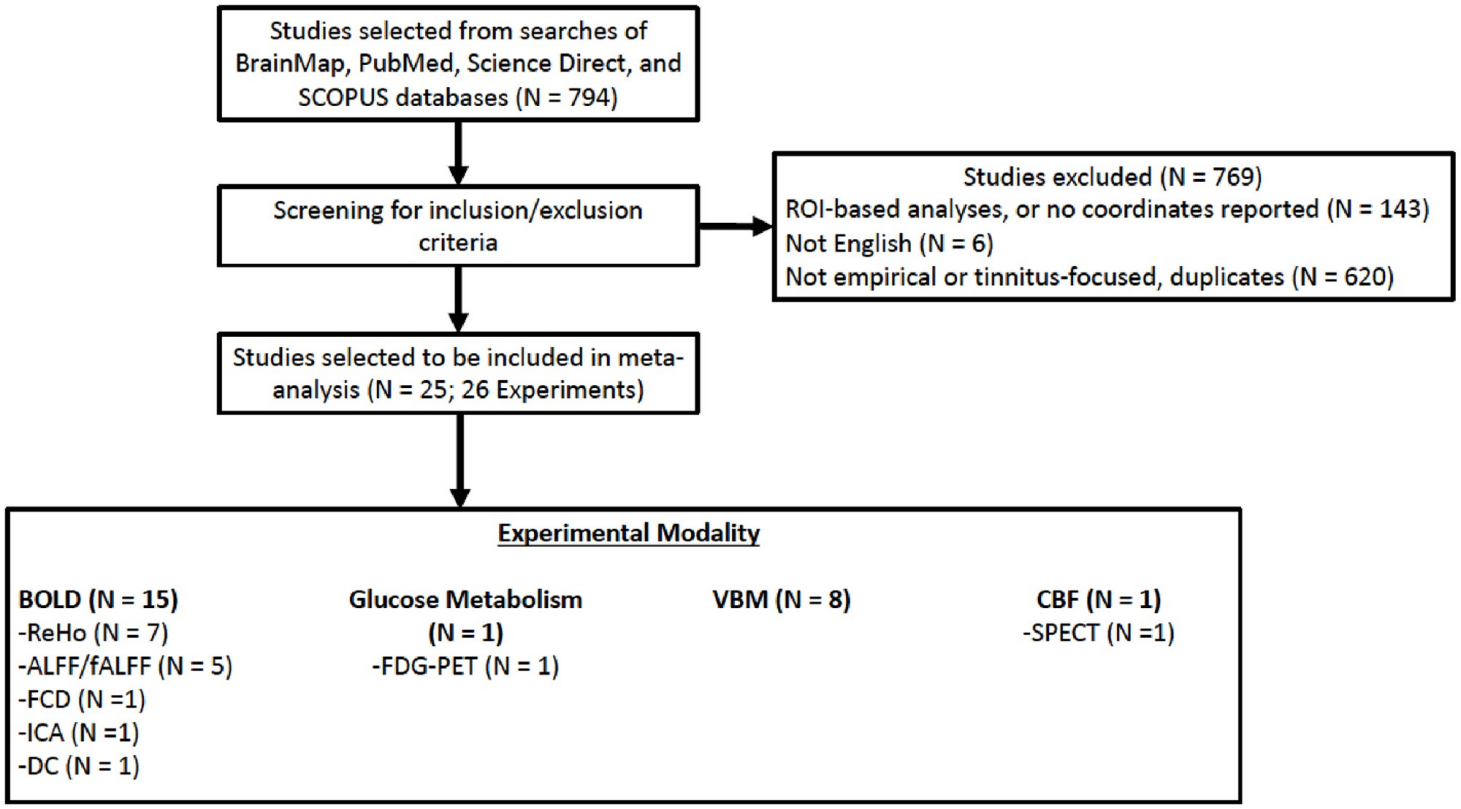
Consort chart of study selection of tinnitus neuroimaging studies. ALFF/fALFF = amplitude of low-frequency fluctuations (ALFF) and fractional ALFF; BOLD = blood oxygen level dependent; DC = degree centrality; FCD = functional connectivity density; FDG-PET = fluorodeoxyglucose PET; ICA = indenpendent components analysis; PET = positron emission tomography; ReHo = Regional homogeneity; SPECT = single-photon emission computed tomography; VBM = voxel-based morphometry.

A literature search of PubMed, BrainMap [10,21,30–34], Scopus, and Science Direct was performed to identify tinnitus VBM and VBP studies, comparing individuals with tinnitus to healthy controls. Trace referencing was also conducted to identify studies with the same criteria. Included studies of gray-matter volume utilized VBM methods. Studies of resting-state VBP included glucose metabolism, amplitude of low frequency fluctuations (ALFF/fALFF), regional homogeneity (ReHo), and regional cerebral blood flow (rCBF). Search terms included: tinnitus; resting-state; brain activity; arterial spin labeling OR ASL; regional homogeneity OR ReHo; glucose metabolism; single photon emission computed tomography OR SPECT; positron emission tomography OR PET; regional cerebral blood flow OR rCBF; gray matter; voxel-based morphometry OR VBM. Any studies that were ambiguous regarding meeting inclusion criteria were screened by a second author. The literature search was completed January 2020. A study selection diagram for this meta-analysis can be seen in Fig 1.

### Study selection criteria pertaining to indices of quality

Selection criteria required that studies be peer-reviewed, English language neuroimaging reports, included application of motion correction, and included participants with unilateral, bilateral, subjective, or pulsatile tinnitus, with any degree of hearing loss. Studies must have compared tinnitus groups to control groups that consisted of participants without any type of tinnitus and must have used voxel-wise whole-brain methods. Studies must have reported results as coordinates using standard reference space: Talairach or Montreal Neurological Institute (MNI). Studies that did not report results in the form of standardized coordinates were excluded from analyses. Data collation was conducted by the first author. The data that support the findings are available in Open Science Framework [35].

### Corrected ALE

The dual threshold CBMA ALE was conducted with cluster-level threshold and family-wise error rate of *p* < .05, 1000 thresholding permutations, and intensity threshold of *p* < .05. The intensity threshold was chosen based on the recommendations for conducting neuroimaging meta-analyses [26]. The cluster-level FWE threshold of *p* < .001 resulted in no effects, and therefore, the cluster-level threshold FWE threshold was expanded to *p* < .05. ALE examines the spatial convergence among previously reported coordinates of the included tinnitus studies and tests the null hypothesis that coordinates are randomly distributed rather than statistically convergent. This approach is blind to magnitude and sign (+/-) and computes the convergence of findings based solely on location. This flexibility allows for ALE to incorporate findings from across imaging modalities [25] to determine the most statistically convergent brain regions related to the disease effects of tinnitus. ALE was computed using GingerALE, [21,30,31] version 3.0 (http://brainmap.org) which simulates random coordinates based on study sizes to simulate noise and increase robustness of results [36].

#### Noise simulation

The current ALE algorithm does not take into account publication biases, in which only significant findings are published, otherwise known as the file drawer effect. Fortunately, Acar et al. [22] developed the fail-safe N method in order to account for publication bias by introducing noise into the ALE algorithm. A modified version of the fail-safe N method [25] was implemented, which introduced 6% noise. Increased noise was introduced until results were no longer significant.

### Uncorrected ALE

Additionally, we implemented a less conservative approach to identify other possible relevant regions impacted by disease effects of tinnitus. This approach did not use statistical methods to correct for multiple comparisons. Thresholds of intensity (*p* < .01) and extent (minimum volume set at 450mm^3^) were implemented for exploratory purposes. A stricter threshold, compared to the dual-threshold method, was used because the recommended threshold of *p* < .05 resulted in over 120 regions, which is not interpretable or meaningful. The more conservative approach that was used also simultaneously limits potential for Type 1 error.

## Results

**Fig 2:**
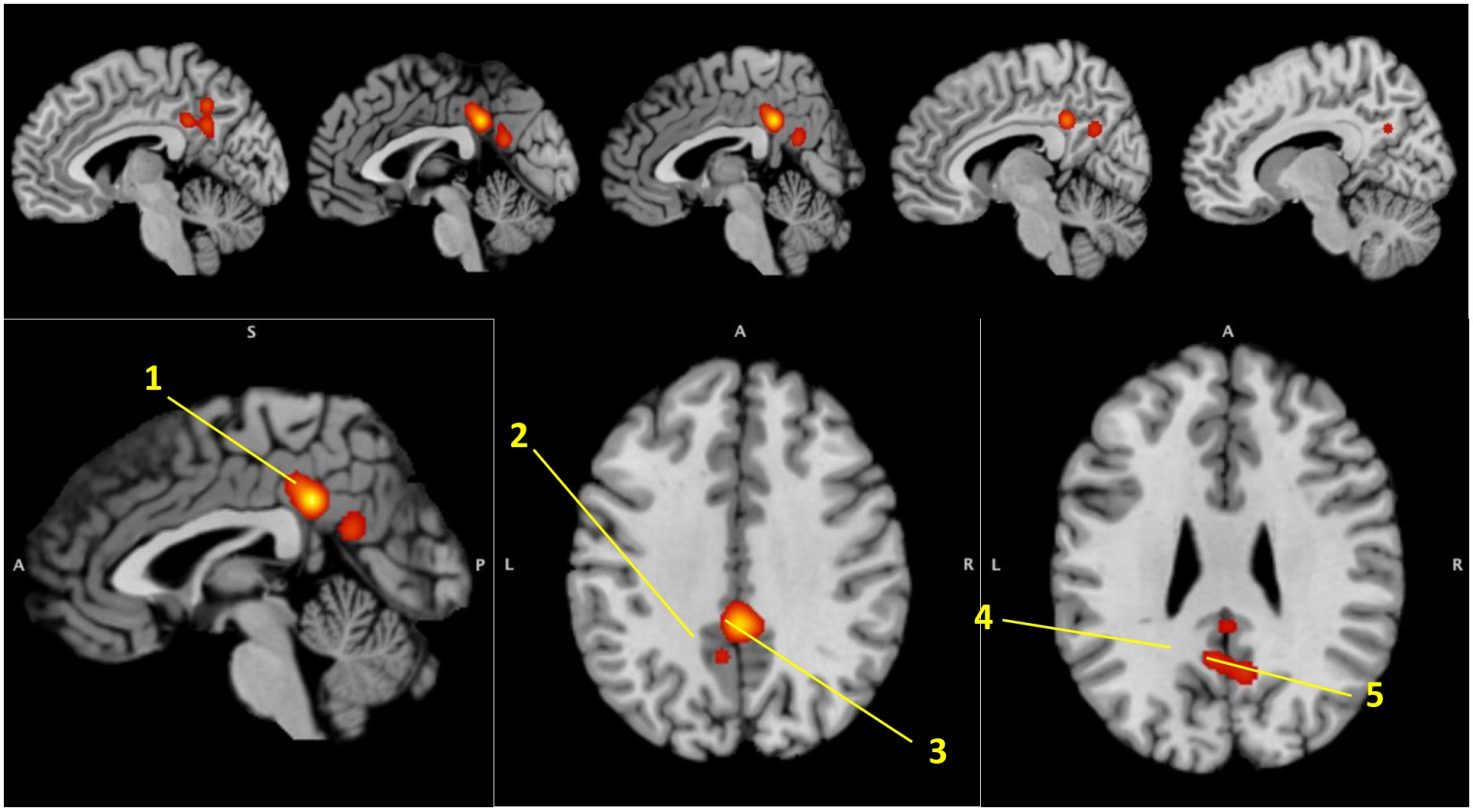
Regions identified by corrected ALE. Note: 1 = Cingulate Gyrus; 2 = Precuneus; 3 = Cingulate Gyrus/Precuneus; 4 = Posterior Cingulate/Precuneus; 5 = Posterior Cingulate/Precuneus.

A total of 25 studies (26 experiments), with 791 participants with tinnitus, were identified for inclusion in this meta-analysis (Table 1). Fig 1 shows the flow diagram of study selection. The all-effects analysis comprised a total of 148 foci from all experiment types. The FWE corrected ALE demonstrated one cluster with five regions of convergence (Fig 2): cingulate gyrus, precuneus, and three regions within the posterior cingulate gyrus (PCG)/precuneus, with a minimum cluster size of 5,728 mm^3^. Coordinates and peak ALE scores from the dual-threshold ALE can be seen in Table 2.

**Table 1:** Studies included in meta-analysis.

Study	Journal	Modality	Patient N	Control N	Mean Age Patient	Mean Age Control	Foci No.	Scanner	Processing Software	Smoothing kernel (mm)	Statistical threshold	MNI or Tal
[37] Boyen et al. (2013)	Hearing Research	VBM	31	24	56	58	9	Philips 3T	SPM5	8	.05 FWE	MNI
[38] Carpenter-Thompson et al. (2014)	Brain Research	BOLD	13	24	54.7	51.4	20	Siemens 3T	SPM8	4	.05 FWE	MNI
[39] Chen et al. (2014)	Neuroimage: Clinical	BOLD-ALFF	31	32	41.9	46.5	7	Siemens 3T	SPM8	4	.05 AlphaSim	MNI
[40] Chen et al. (2015)	Neural Plasticity	BOLD-Reho	29	30	40.9	46.2	5	Siemens 3T	SPM8	4	.01 AlphaSim	MNI
[41] Chen et al. (2016)	Frontiers in Aging Neuroscience	BOLD-DC	24	22	50.8	44.7	5	Philips 3T	SPM8	6	.01 AlphaSim	MNI
[42] Gentil et al. (2019)	Trends in Hearing	BOLD-Reho	19	16	63	59	1	Siemens 3T	SPM12	6	.005	MNI
[43] Geven et al. (2014)	Neuroscience	FDG-PET	20	19	51	50.8	2	Siemens	SPM5	8	.001 Uncorrected	MNI
[44] L. Han et al. (2015)	Neuroscience	FCD	32	32	37.1	38.5	16	GE 3T	SPM8	6	.05 AlphaSim	MNI
[45] Lv Han et al. (2015)	Progress in Neuro-Psychopharmacology and Biological Psychiatry	BOLD-ReHo/ALFF	34	34	37.9	39.5	14	GE 3T	SPM8	4	.05 Monte Carlo	MNI
[46] Han et al. (2014)	Neural Plasticity	BOLD-ALFF	42	42	37.2	37	11	GE 3T	SPM8	4	.01 Monte Carlo	MNI
[47] Han et al. (2018)	Neuroradiology	BOLD-ReHo	25	25	44.7	44	5	Siemens 3T	SPM8	5	.05 AlphaSim	MNI
[27] Husain et al. (2011)	Brain Research	VBM	8	18	56.1	51.4	5	GE 3T	SPM5	8	.001 Uncorrected	MNI
[48] Laureano et al. (2014)	PloS One	SPECT	20	17	42.9	41.4	1	GE	SPM8	8	.05 FWE	MNI
[49] Leaver et al. (2012)	Frontiers in Systems Neuroscience	VBM	23	21	47.4	49	3	Siemens 3T	SPM8	6	.05 Uncorrected	MNI
[50] Leaver et al. (2016)	Human Brain Mapping	BOLD-ICA	21	19	47.3	48.9	5	Siemens 3T	Brain Voyager	6	.0005 Uncorrected	Tal
[51] Liu et al. (2018)	Neural Plasticity	VBM	24	24	34.9	35.3	7	GE 3T	SPM8	6	.001 Uncorrected	MNI
[52] Lv et al. (2017)	Hearing Research	BOLD-ReHo	45	45	37.3	37.2	4	GE 3T	SPM8	4	.01 FDR	MNI
[53] Maudoux et al. (2012)	PloS One	BOLD-ICA	13	15	52	51	17	Siemens 3T	Brain Voyager	8	.05 FDR	Tal
[54] Melcher et al. (2013)	Hearing Research	VBM	24	24	46.9	45.8	1	Siemens 3T	SPM8	8	.001 Uncorrected	MNI
[55] Mühlau et al. (2006)	Cerebral Cortex	VBM	28	28	40	39	1	Siemens 1.5T	SPM2	8	.05 FDR	MNI
[56] Schmidt et al. (2018)	Brain Research	VBM	15 13 18 19	13 15 - -	55.1 57.6 52.33 51.2	48.4 52.9 NA NA	11	Siemens 3T	SPM12	10	.05 FWE	MNI
[57] Seydell-Greenwald et al. (2012)	Brain Research	BOLD	20	20	47	49	2	Siemens 3T	Brain Voyager	6	.005 Uncorrected	Tal
[58] Vanneste et al., (2015)	PloS One	VBM	154	-	50.24	NA	9	Siemens 3T	SPM8	8	.001 Uncorrected	MNI
[59] Yang et al. (2014)	Journal of Otology	BOLD-ReHo	18	20	43	42	2	Philips 3T	SPM5	NA	.05 FWE	MNI
[60] Zhou et al. (2019)	Frontiers in Neuroscience	BOLD-ReHo/FALFF	28	31	41.2	45.4	6	Philips 3T	SPM8	4	.001 AlphaSim	MNI

**Table 2:** Identified regions from corrected ALE.

Region Name	MNI Coordinate (x, y, z)	BA	Peak ALE Score	Peak Z Score	Fail-Safe N (%)
**ALE Results**					
Cingulate Gyrus	2, -42, 32	31	.022	5.043	11%>FSN>6%
Precuneus	-6, -54, 42	7	.010	3.090	11%>FSN>6%
Cingulate Gyrus/Precuneus	-6, -54, 30	31	.009	3.023	11%>FSN>6%
Posterior Cingulate/Precuneus	0, -58, 22	23	.009	2.890	11%>FSN>6%
Posterior Cingulate/Precuneus	8, -60, 28	31	.008	2.507	11%>FSN>6%

Note: ALE = Activation Likelihood Estimate; BA = Brodmann Area; MNI = Montreal Neurological Institute.

The fail-safe N method assessed the robustness of the corrected ALE findings, which accounted for unpublished findings. A total of 6% noise was added to the meta-analysis, and results remained consistent regarding the significant cluster and five regions of convergence described above. However, when 11% of added noise was added to the meta-analytic data, these results were not replicated.

**Fig 3:**
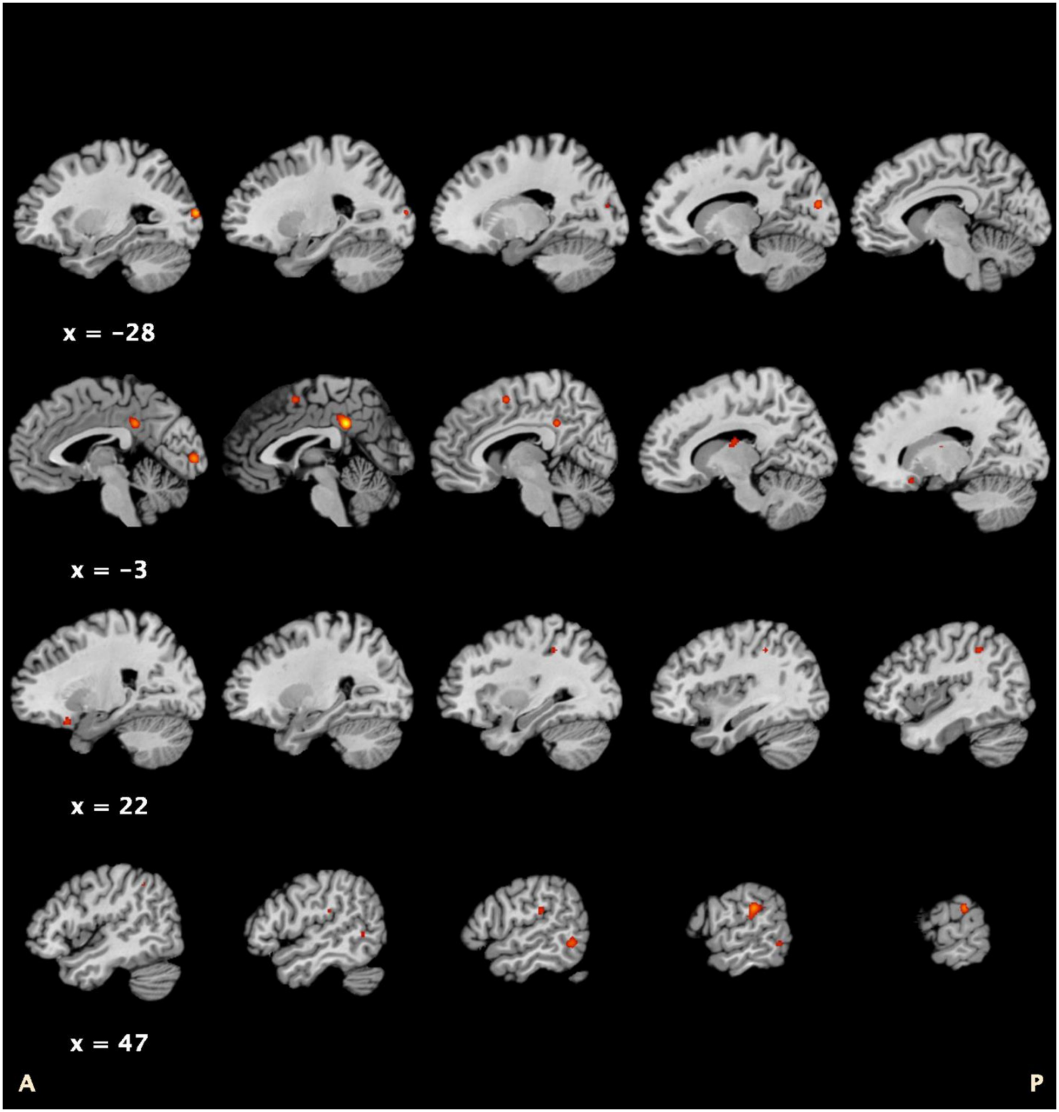
Regions identified by uncorrected ALE. Note: Areas in color represent the 15 regions identified by the uncorrected ALE, p < .05.

After implementation of an uncorrected ALE, results demonstrated 15 regions across 10 clusters. The first cluster contained the inferior parietal lobe and insula, while the second cluster replicated the findings from the corrected ALE. This particular cluster contained one region: the cingulate gyrus. Additional regions within the remaining nine clusters included the middle temporal gyrus, lingual gyrus, middle occipital gyrus, cuneus, medial frontal gyrus, subcallosal gyrus, and thalamus. Fig 3 shows the clusters that resulted from the single-threshold ALE. Coordinates and peak ALE scores from the single-threshold ALE can be seen in Table 3. The data that support all findings are available in Open Science Framework at osf.io [35].

**Table 3:** Identified regions from uncorrected ALE.

Region Name	MNI Coordinate (x, y, z)	BA	Peak ALE Score	Peak Z Score
**ALE Results**				
R Inferior Parietal Lobule	64, -30, 24	40	.018	4.459
R Insula	56, -28, 22	13	.010	3.062
R Superior Temporal Gyrus	60, -40, 24	13	.009	2.831
L Cingulate Gyrus	2, -42, 32	31	.022	5.043
R Middle Temporal Gyrus	58, -54, -8	37	.013	3.577
R Middle Temporal Gyrus	54, -56, 0	37	.009	3.002
L Lingual Gyrus	-2, -94, 2	18	.015	4.005
R Inferior Parietal Lobule	42, -42, 42	40	.010	3.126
R Precuneus	34, -40, 42	7	.009	3.008
R Inferior Parietal Lobule	46, -48, 44	40	.008	2.604
L Middle Occipital Gyrus	-26, -96, 10	18	.018	4.459
L Cuneus	-14, -88, 16	17	.013	3.632
R Middle Frontal Gyrus	4, 4, 54	6	.013	3.655
R Subcallosal Gyrus	22, 18, -22	47	.010	3.154
R Thalamus	10, 14, -10	-	.010	3.148

Note: ALE = Activation Likelihood Estimate; BA = Brodmann Area; L = Left; MNI = Montreal Neurological Institute; R = Right.

## Discussion

### Summary

The current findings represent the disease effects of tinnitus in grey matter, glucose metabolism, and blood flow. Two ALEs were implemented with different levels of statistical rigor: corrected for multiple comparisons and uncorrected. The cluster-level inference ALE with family-wise error (FWE) rate was more stringent and demonstrated one cluster with five regions related to the disease effects of tinnitus. These regions included the posterior cingulate gyrus/precuneus and cingulate gyrus. Moreover, these regions remained significant in relation to tinnitus disease-effects after introducing 6% added noise, which accounts for negative unpublished findings. The second approach did not implement a statistical correction for multiple comparisons, which was less stringent, and identified 15 additional regions across 10 clusters. These regions included the cingulate gyrus, occipital temporal gyrus, lingual gyrus, middle occipital gyrus, cuneus, medial frontal gyrus, subcallosal gyrus, and thalamus.

Findings from the cluster-level inference ALE with FWE serve as an out-of-sample replication of previous tinnitus neuroimaging studies of resting-state functional connectivity. Resting-state functional connectivity measures temporal correlations of spontaneous BOLD signals across brain regions [61] to identify disease-related networks. For example, Schmidt et al. [62] found that individuals with tinnitus exhibited decreased connectivity within the left precuneus, left precentral gyrus, and left cerebellum, compared to individuals with hearing loss and no tinnitus. Additional out-of-sample replications are observed by the findings from the ALE without FWE regarding the auditory dorsal attention network (DAN).

Our results demonstrate consistent disease-related effects of tinnitus across a heterogeneous population that varies in tinnitus sounds, loudness, laterality, and duration of tinnitus. Other medical and psychological comorbidities, such as head injury, hearing loss, depression, posttraumatic stress disorder, and anxiety, were not controlled in the current study; nor were the data acquisition techniques and data analytic approaches. Therefore, it is suggested that the identified regions from this meta-analysis, particularly from the cluster-level inference ALE with FWE, are invariant and shared across the spectrum of tinnitus patients. Findings are explained in the context of the resting-state network [28,63] for which specific regions are most identified with.

### Corrected ALE

#### Default mode network

The cluster-level inference ALE with FWE demonstrated the disease-effects of tinnitus occur within the posterior cingulate cortex (PCC), cingulate gyrus, and precuneus, which play a central role within the default mode network [64–68]. It is important to note that the studies that contributed to the significant clusters found by the ALE indicated heightened activation for those with tinnitus, when compared to controls [38,44–46,52,60]. Interestingly, there were no VBM studies that contributed to these results. The default mode network (DMN) is a “task negative network” [32], meaning that it is more highly activated at “rest;” however, recent research suggests a more complicated pattern, specifically within the PCC [69]. In addition to task-free states, the PCC is likely involved in ongoing experiences, most notably self-generated thoughts such as daydreaming, the recollection of autobiographical information, and future planning, particularly of a social nature [64,70]. A recent meta-analytic study demonstrated the significant role of the PCC in domains of cognition, and particularly in attention, language, and memory [71]. Moreover, the same study differentiated between the dorsal and ventral PCC (dPCC and vPCC, respectively) and found that the dPCC was highly co-activated with regions associated with consciousness and awareness of internal bodily sensations, hereafter called interoceptive awareness. Additionally, the vPCC was found to co-activate with regions associated with self-awareness. Related to tinnitus and the results of the current study, alteration of the PCC may hinder tinnitus patients from appropriately directing their attention away from irrelevant noise sources, and instead, increase individuals’ attention and awareness to the tinnitus percept. Therefore, therapies should aim to provide individuals with tinnitus the techniques to increase their capability of re-directing their focus from their tinnitus and instead to the present moment and engaging in value-based activities, such as mindfulness-based cognitive therapy [72]. Future psychotherapies and neuromodulatory approaches should aim to decrease the activation within the DMN; however, additional research is warranted concerning the connectomic properties of the DMN among tinnitus patients.

It is important to note that the cluster-level inference CBMA ALE with FWE did not detect any regions within the auditory RSN. Instead, the superior, middle, and inferior temporal gyrus was detected by the single-threshold CBMA ALE, discussed later. Past and current perspectives on tinnitus have heavily relied on the assumption that auditory regions are most implicated for the genesis and maintenance of tinnitus. While it is certainly understandable to conceptualize tinnitus as an auditory disorder, a detriment of this approach includes a hyper-focus on the auditory RSN, and therefore, a concurrent and serious neglect of other significant regions and networks.

Our results offer strong support for the paradigm shift in the field of tinnitus regarding the specific neurobiological disease-related effects of the disorder [64,73]. It is especially noteworthy that the regions within the DMN, found to be significantly associated with tinnitus, survived a fail-safe N correction for unreported negative finding, adding up to 6% random noise. Therefore, even when accounting for unpublished results, the corrected ALE demonstrates results with an acceptable amount of added noise [74]. Extant research, however, is heavily influenced by the notion that tinnitus is an auditory disorder; and therefore, significant differences in brain structure and function should be found within the auditory network (AUD). Song et al. [12] conducted a TA tinnitus meta-analysis among 10 studies, in which 6 of the studies did not have a control group, and two of the remaining studies used sound as the task dimension. Beyond the criticism related to insufficient studies to perform CBMA ALE [26], this study is overly reliant on sound tasks that undoubtedly increases potential for Type I error, specifically related to the auditory network. Moreover, the field has neglected findings that have demonstrated significant effects of tinnitus, beyond the AUD. One glaring example of this neglect in tinnitus neuroimaging research is found in Farhadi et al. [75], in which authors focus on results solely related to temporal regions despite the clear implication of the cingulate gyrus. In order to progress the field of tinnitus research, investigators must recognize the effects of regions across canonical networks, and rely less heavily on past assumptions.

### Uncorrected ALE

In addition to the rigorous meta-analysis discussed above, an uncorrected meta-analysis was conducted to detect all possible alterations that might represent disease-effects of tinnitus. This less conservative approach has a greater probability of Type I error; however, due to the relative novelty of tinnitus neuroimaging research, it is important to identify a larger set of regions that may otherwise be overlooked. In this sense, future research can determine whether these regions are significantly associated with tinnitus. The regions identified beyond what was found by the ALE can be discussed in relation to two RSNs: auditory (AUD) and cognitive processes.

#### Auditory

Disease-effects of tinnitus within the AUD were discovered when the uncorrected ALE was implemented. Specifically, the middle temporal gyrus and sub-gyral (non-cortical) regions within AUD demonstrated alterations. The middle temporal gyrus (MTG) is an extra-primary cortical component of the auditory network strongly implicated in language and of semantic processing, in particular [76,77]. Individuals experiencing hearing loss with and without tinnitus demonstrate increased gray matter in the MTG when compared to individuals without tinnitus or hearing loss [37]. Therefore, the MTG may be more related to hearing loss, rather than tinnitus. However, alterations within the right MTG have also been demonstrated among tinnitus sufferers both as structural differences and in spontaneous neural activity [78]. Dysregulation within the MTG may be a source of the tinnitus percept, while other regions of the brain may play a role in the loudness and emotional responses associated with tinnitus [79]. Investigation of the functional and structural connectivity between the MTG and other significant regions identified by the current study will likely shed light on specific neurobiological causes of tinnitus and related distress.

#### Cognitive processes

The IPL is a hub of the dorsal attention network (DAN), which is associated with cognitive abilities and top-down processing [80]. Previous research supports the role of the DAN in tinnitus [62,81], and, more recently, increased connectivity between the DAN and precuneus (DMN) has been shown to reflect tinnitus duration and severity [82]. Authors of these studies suggest that the DAN and DMN may not be appropriately suppressed at rest or during tasks, respectively. The DAN may be active “at-rest” and while “task-positive,” representing lack of control away from tinnitus sensation, and perhaps, negative attributions toward tinnitus, even while external noises mask the tinnitus percept. The medial frontal gyrus (MFG) was also found to be a significant brain region based. The MFG (BA 10) is a region associated with executive functioning, which includes motor planning, decision-making, abilities to hold ideas in working memory, as well as switching cognitive sets [83]. Studies have demonstrated decreased grey matter volume within the MFG among individuals with schizophrenia [84], who often have significant deficits in executive functioning tasks. For those with tinnitus, and in relation to the results that implicate the IPL, individuals may have difficulty switching their attention away from tinnitus sounds. As causation cannot be determined, it is also possible that the alterations of the MFG may be due to distractibility related to tinnitus.

#### Neural plasticity

Often referred to as a plasticity disorder [85–87], tinnitus may occur due to compensatory processes, involving deafferentation and increased spontaneous firing of neurons beyond the auditory pathway [88], causing alterations observed in this meta-analysis. It is suggested that in response to acoustic trauma, individuals who develop tinnitus experience significantly greater structural and functional changes within the posterior cingulate and precuneus, compared to individuals who may have experienced similar acoustic events and did not develop tinnitus. Such changes may also reflect neuroplasticity occurring after onset of chronic tinnitus as individuals adapt to the condition. Longitudinal epidemiologic studies that implement neuroimaging techniques may be able to ascertain the chronological order of changes within these brain regions that can predict development of tinnitus. However, since imaging is conducted during a discrete timeframe, observed neurobiological differences may be a function of events prior to the onset of tinnitus, or the habituation to chronic tinnitus.

### Future directions

Future studies should aim to more fully characterize, structurally and functionally, altered brain regions indicative of disease-related effects of tinnitus, without the reliance on past assumptions related to the auditory network. These results provide compelling evidence that a paradigm shift is necessary in the field of tinnitus neuroimaging research. Investigators must recognize the effects from specific regions across canonical networks beyond the auditory resting-state network.

Functional modeling may help distinguish the relationship among the regions identified in this meta-analysis, including direct and indirect pathways involved in tinnitus generation, persistence, tolerance, bothersomeness, and habituation. Co-authors of this study aim to identify the functional pathways of tinnitus. By doing so, neuromodulatory therapies may become more refined and tailored to individuals’ needs regarding comorbid diagnoses and current health conditions.

### Caveats

It is feasible that improvement of the spatial resolution of existing tools may lead to the identification of additional altered regions associated with tinnitus. Additionally, this meta-analysis did not control for demographic variables, psychological comorbidities, head injury, hearing loss, or differences in tinnitus percept or related distress. Future neuroimaging studies may account for these differences to map tinnitus in relation to specific comorbidities. Moreover, although ALE found significant regions within clusters, precise areas (e.g., dorsal/ventral PCC) are less well identified.

## Supporting information

S1 Checklist PRISMA 2020 checklist. (DOCX)
