# Supplementary material for: Invariant structural and functional brain regions associated with tinnitus: A meta-analysis
Source: PLoS One. 2022 Oct 18;17(10):e0276140. doi: 10.1371/journal.pone.0276140 (PMC9578602; doi:10.1371/journal.pone.0276140)
Supplement: S1 Checklist — (DOCX) [file pone.0276140.s001.docx]

| **Section and Topic** | **Item #** | **Checklist item** | **Location where item is reported** |
| --- | --- | --- | --- |
| **TITLE** | | |  |
| Title | 1 | Identify the report as a systematic review.  In a meta-analysis that examines the convergence of brain regions associated with neurodegeneration due to tinnitus, the authors identify the report as a meta-analysis:  “Invariant structural and functional brain regions associated with tinnitus: A meta-analysis | Title Page, Page 1 |
| **ABSTRACT** | | |  |
| Abstract | 2 | See the PRISMA 2020 for Abstracts checklist.  In a meta-analysis that examines available data of voxel-based physiology and voxel-based morphometry studies related to differences between individuals with tinnitus and controls, the authors describe how many studies were included in the meta-analysis, the methodology of the meta-analytic approach, results, and comment on the brain regions most associated with disease-effects of tinnitus.  “Tinnitus is a common, functionally disabling condition of often unknown etiology. Neuroimaging research to better understand tinnitus is emerging but remains limited in scope. Voxel-based physiology (VBP) studies detect tinnitus-associated pathophysiology by group-wise contrast (tinnitus vs controls) of resting-state indices of hemodynamics, metabolism, and neurovascular coupling. Voxel-based morphometry (VBM) detects tinnitus-associated neurodegeneration by group-wise contrast of structural MRI. Both VBP and VBM studies routinely report results as atlas-referenced coordinates, suitable for coordinate-based meta-analysis (CBMA). Here, 17 resting-state VBP and 8 VBM reports of tinnitus-associated regional alterations were meta-analyzed using activation likelihood estimation (ALE). Acknowledging the need for data-driven insights, ALEs were performed at two levels of statistical rigor: corrected for multiple comparisons and uncorrected. The corrected ALE applied cluster-level inference thresholding by intensity (z-score > 1.96; p < 0.05) followed by family-wise error correction for multiple comparisons (p < .05, 1000 permutations) and fail-safe correction for missing data. The corrected analysis identified one significant cluster comprising five foci in the posterior cingulate gyrus and precuneus, that is, not within the primary or secondary auditory cortices. The uncorrected ALE identified additional regions within auditory and cognitive processing networks. Taken together, tinnitus is likely a dysfunction of regions spanning multiple canonical networks that may serve to increase individuals’ interoceptive awareness of the tinnitus sound, decrease capacity to switch cognitive sets, and prevent behavioral and cognitive attention to other stimuli. It is noteworthy that the most robust tinnitus-related abnormalities are not in the auditory system, contradicting collective findings of task-activation literature in tinnitus.” | Abstract, Page 2 |
| **INTRODUCTION** | | |  |
| Rationale | 3 | Describe the rationale for the review in the context of existing knowledge.  In a meta-analysis that combines both structural and functional neuroimaging data, the authors state that it remains important to combine data considering that the activation likelihood estimate assesses the spatial proximity of reported coordinates.  “Although ALE CBMA was originally designed for task-activation meta-analysis and has been most extensively used for single-modality, (see BrainMap.org/pubs), this is not an intrinsic limitation of the ALE method. Rather, ALE assesses the spatial proximity of reported coordinates against a null hypothesis of a random distribution of the same volume and quality of data. This modality agnosticism allows ALE the flexibility to integrate findings across imaging methods (Garrett et al., 2019; Gray et al., 2020), if to do so is logically appropriate. In the present study, we combined resting-state VBP studies and VBM studies contrasting persons with tinnitus to healthy controls for a comprehensive assessment of tinnitus-related gray-matter alterations.” | Introduction, Page 5 |
| Objectives | 4 | Provide an explicit statement of the objective(s) or question(s) the review addresses.  In a meta-analysis that examines the convergence of brain regions related to the disease-effects of tinnitus, authors explain that the goal was to demonstrate that non-auditory regions will be significantly associated with tinnitus.  “The overall goal of the present study was to identify brain regions exhibiting tinnitus-related functional and structural alterations in the absence of task performance by applying CBMA to the VBP and VBP literatures. The null hypothesis of ALE CBMA is spatial non-convergence (i.e. a random data distribution). The hypothesis of the investigators was that this task-free approach would demonstrate abnormalities outside the confines of the auditory system and provide new, data-driven insights into the pathophysiology of tinnitus (Husain et al., 2011; 2014; 2019).” |  |
| **METHODS** | | |  |
| Eligibility criteria | 5 | Specify the inclusion and exclusion criteria for the review and how studies were grouped for the syntheses.  In a tinnitus neuroimaging meta-analysis, authors describe the eligibility for studies to be included in the analyses.  “Selection criteria required that studies be peer-reviewed, English language neuroimaging reports, included application of motion correction, and included participants with unilateral, bilateral, subjective, or pulsatile tinnitus, with any degree of hearing loss. Studies must have compared tinnitus groups to control groups that consisted of participants without any type of tinnitus and must have used voxel-wise whole-brain methods. Studies must have reported results as coordinates using standard reference space: Talairach or Montreal Neurological Institute (MNI). Studies that did not report results in the form of standardized coordinates were excluded from analyses.” | Methods, Page 7 |
| Information sources | 6 | Specify all databases, registers, websites, organisations, reference lists and other sources searched or consulted to identify studies. Specify the date when each source was last searched or consulted.  In a tinnitus neuroimaging meta-analysis, authors explicitly state the databases used to search for relevant articles.  “A literature search of PubMed, BrainMap (Eickhoff et al., 2012; Eickhoff et al., 2009; Fox et al., 2005; Fox & Lancaster, 2002; Laird et al., 2005; Turkeltaub et al., 2012; Vanasse et al., 2018), Scopus, and Science Direct was performed to identify tinnitus VBM and VBP studies, comparing individuals with tinnitus to healthy controls. Trace referencing was also conducted to identify studies with the same criteria.” | Methods, Page 6 |
| Search strategy | 7 | Present the full search strategies for all databases, registers and websites, including any filters and limits used.  Authors in a meta-analysis of tinnitus neuroimaging studies included the full search strategy.  “Search terms included: tinnitus; resting-state; brain activity; arterial spin labeling OR ASL; regional homogeneity OR ReHo; glucose metabolism; single photon emission computed tomography OR SPECT; positron emission tomography OR PET; regional cerebral blood flow OR rCBF; gray matter; voxel-based morphometry OR VBM.” | Methods, Page 6 |
| Selection process | 8 | Specify the methods used to decide whether a study met the inclusion criteria of the review, including how many reviewers screened each record and each report retrieved, whether they worked independently, and if applicable, details of automation tools used in the process.  In a tinnitus neuroimaging meta-analysis, one primary author was responsible for deciding whether studies met inclusion criteria. Articles with ambiguous inclusion criteria were sent to a second author.  “Any studies that were ambiguous regarding meeting inclusion criteria were screened by a second author.” | Methods, Page 6 |
| Data collection process | 9 | Specify the methods used to collect data from reports, including how many reviewers collected data from each report, whether they worked independently, any processes for obtaining or confirming data from study investigators, and if applicable, details of automation tools used in the process.  In a tinnitus neuroimaging meta-analysis, the primary author collected the data.  “Data collation was conducted by the first author.” | Methods, Page 7 |
| Data items | 10a | List and define all outcomes for which data were sought. Specify whether all results that were compatible with each outcome domain in each study were sought (e.g. for all measures, time points, analyses), and if not, the methods used to decide which results to collect.  In a tinnitus neuroimaging meta-analysis, coordinates from studies were used for the data analysis.  “Studies must have reported results as coordinates using standard reference space: Talairach or Montreal Neurological Institute (MNI).” | Methods, Page 7 |
|  | 10b | List and define all other variables for which data were sought (e.g. participant and intervention characteristics, funding sources). Describe any assumptions made about any missing or unclear information.  N/A |  |
| Study risk of bias assessment | 11 | Specify the methods used to assess risk of bias in the included studies, including details of the tool(s) used, how many reviewers assessed each study and whether they worked independently, and if applicable, details of automation tools used in the process.  In a tinnitus neuro-imaging meta-analysis, authors used noise simulation to address risk of bias.  “The current ALE algorithm does not take into account publication biases, in which only significant findings are published, otherwise known as the file drawer effect. Fortunately, Acar et al. (2018) developed the fail-safe N method in order to account for publication bias by introducing noise into the ALE algorithm. A modified version of the fail-safe N method (Gray et al., 2020) was utilized, which introduced 6% noise. Increased noise was introduced until results were no longer significant.” | Methods, Page 8 |
| Effect measures | 12 | Specify for each outcome the effect measure(s) (e.g. risk ratio, mean difference) used in the synthesis or presentation of results.  N/A |  |
| Synthesis methods | 13a | Describe the processes used to decide which studies were eligible for each synthesis (e.g. tabulating the study intervention characteristics and comparing against the planned groups for each synthesis (item #5)).  N/A |  |
|  | 13b | Describe any methods required to prepare the data for presentation or synthesis, such as handling of missing summary statistics, or data conversions.  N/A |  |
|  | 13c | Describe any methods used to tabulate or visually display results of individual studies and syntheses.  In a tinnitus neuro-imaging meta-analysis, authors show all studies that were included in the analyses.  “See Table 1.” | Results, Page 8 |
|  | 13d | Describe any methods used to synthesize results and provide a rationale for the choice(s). If meta-analysis was performed, describe the model(s), method(s) to identify the presence and extent of statistical heterogeneity, and software package(s) used.  In a tinnitus neuro-imaging meta-analysis, authors describe using the activation likelihood estimate, with the statistical software package GingerALE.  “The dual threshold CBMA ALE was conducted with cluster-level threshold and family-wise error rate of p < .05, 1000 thresholding permutations, and intensity threshold of p < .05. ALE examines the spatial convergence among previously reported coordinates of the included tinnitus studies and tests the null hypothesis that coordinates are randomly distributed rather than statistically convergent. ALE was computed using GingerALE, (Eickhoff et al., 2012; Eickhoff et al., 2009; Turkeltaub et al., 2012) version 3.0 (http://brainmap.org) which simulates random coordinates based on study sizes to simulate noise and increase robustness of results (Samartsidis et al., 2017).” | Methods, Page 7 |
|  | 13e | Describe any methods used to explore possible causes of heterogeneity among study results (e.g. subgroup analysis, meta-regression).  N/A |  |
|  | 13f | Describe any sensitivity analyses conducted to assess robustness of the synthesized results.  N/A |  |
| Reporting bias assessment | 14 | Describe any methods used to assess risk of bias due to missing results in a synthesis (arising from reporting biases).  In a tinnitus neuroimaging meta-analysis, authors used the noise simulation Fail-Safe N method to simulate noise.  “The current ALE algorithm does not take into account publication biases, in which only significant findings are published, otherwise known as the file drawer effect. Fortunately, Acar et al. (2018) developed the fail-safe N method in order to account for publication bias by introducing noise into the ALE algorithm. A modified version of the fail-safe N method (Gray et al., 2020) was utilized, which introduced 6% noise. Increased noise was introduced until results were no longer significant.” | Methods, Page 8 |
| Certainty assessment | 15 | Describe any methods used to assess certainty (or confidence) in the body of evidence for an outcome.  In a tinnitus neuro-imaging meta-analysis, authors conducted a secondary ALE, which replicated the first ALE and provided additional results.  “Additionally, we implemented a less conservative approach to identify other possible relevant regions impacted by disease effects of tinnitus. This approach did not utilize statistical methods to correct for multiple comparisons. Thresholds of intensity (p < .01) and extent (minimum volume set at 450mm3) were implemented for exploratory purposes, while simultaneously limiting potential for Type 1 error.” | Methods, Page 8 |
| **RESULTS** | | |  |
| Study selection | 16a | Describe the results of the search and selection process, from the number of records identified in the search to the number of studies included in the review, ideally using a flow diagram.  In a tinnitus neuro-imaging meta-analysis, the selection process and results of the number of records identified were explained.  “A total of 25 studies (26 experiments), with 791 participants with tinnitus, were identified for inclusion in this meta-analysis (Table 1). Figure 1 shows the flow diagram of study selection. The all-effects analysis comprised a total of 148 foci from all experiment types.” | Results, Page 8 |
|  | 16b | Cite studies that might appear to meet the inclusion criteria, but which were excluded, and explain why they were excluded.  In a tinnitus neuro-imaging meta-analysis, authors provide a Figure that describes how many studies were excluded and the reasons for the exclusionary decision.  “A study selection diagram for this meta-analysis can be seen in Figure 1.” | Methods, Page 7 |
| Study characteristics | 17 | Cite each included study and present its characteristics.  In a tinnitus neuro-imaging meta-analysis, each included study is cited and presented in Table 1.  “See Table 1.” | Results, Page 8 |
| Risk of bias in studies | 18 | Present assessments of risk of bias for each included study.  Risk of bias is presented as an argument for the reason to conduct a tinnitus neuroimaging meta-analysis.  “Acknowledging the limited volume of the quantitative, coordinate-reporting literature in tinnitus and the necessity for data-driven etiological insights, CBMAs were performed at two levels of statistical rigor: confirmatory and exploratory. The more conservative approach applied two statistical thresholds: (1) intensity (p < 0.05) and extent thresholding (> 4,500 mm3) at the voxel level; and, (2) a correction for multiple comparisons at the cluster-forming level using (family-wise error rate, 1,000 permutations, p < 0.05). Additionally, the fail-safe correction for missing data (publication bias) was applied (Acar et al., 2018; Gray et al., 2020). The less conservative approach applied only voxel-wise thresholds, with no corrections for multiple comparisons or missing data. The intent of this exploratory analysis was to probe the available data as deeply as possible and thereby simulate hypothesis generation as well as to identify candidate nodes for network analyses.” | Introduction, Pages 5-6 |
| Results of individual studies | 19 | For all outcomes, present, for each study: (a) summary statistics for each group (where appropriate) and (b) an effect estimate and its precision (e.g. confidence/credible interval), ideally using structured tables or plots.  In a tinnitus neuroimaging meta-analysis, authors report on the brain coordinates that were significant, as well as showcase these results in tables and figures.  “The FWE corrected ALE demonstrated one cluster with five regions of convergence (Figure 2): cingulate gyrus, precuneus, and three regions within the posterior cingulate gyrus (PCG)/precuneus. Coordinates and peak ALE scores from the dual-threshold ALE can be seen in Table 2.” | Results, Page 8 |
| Results of syntheses | 20a | For each synthesis, briefly summarise the characteristics and risk of bias among contributing studies.  In a tinnitus neuroimaging meta-analysis, authors reported results of the fail-safe N procedure, which demonstrated robustness of results.  “The fail-safe N method assessed the robustness of the corrected ALE findings, which accounted for unpublished findings. A total of 6% noise was added to the meta-analysis, and results remained consistent regarding the significant cluster and five regions of convergence described above. However, when 11% of added noise was added to the meta-analytic data, these results were not replicated.” | Results, Pages 8-9 |
|  | 20b | Present results of all statistical syntheses conducted. If meta-analysis was done, present for each the summary estimate and its precision (e.g. confidence/credible interval) and measures of statistical heterogeneity. If comparing groups, describe the direction of the effect.  In a tinnitus neuroimaging meta-analysis, authors reported results based on a FWE corrected ALE, with fail-safe N procedures, as well as an uncorrected ALE.  “The FWE corrected ALE demonstrated one cluster with five regions of convergence (Figure 2): cingulate gyrus, precuneus, and three regions within the posterior cingulate gyrus (PCG)/precuneus. Coordinates and peak ALE scores from the dual-threshold ALE can be seen in Table 2.  The fail-safe N method assessed the robustness of the corrected ALE findings, which accounted for unpublished findings. A total of 6% noise was added to the meta-analysis, and results remained consistent regarding the significant cluster and five regions of convergence described above. However, when 11% of added noise was added to the meta-analytic data, these results were not replicated.  After implementation of an uncorrected ALE, results demonstrated 15 regions across 10 clusters. The first cluster contained the inferior parietal lobe and insula, while the second cluster replicated the findings from the corrected ALE. This particular cluster contained one region: the cingulate gyrus. Additional regions within the remaining nine clusters included the middle temporal gyrus, lingual gyrus, middle occipital gyrus, cuneus, medial frontal gyrus, subcallosal gyrus, and thalamus. Figure 3 shows the clusters that resulted from the single-threshold ALE. Coordinates and peak ALE scores from the single-threshold ALE can be seen in Table 3.” | Results, Pages 8-9 |
|  | 20c | Present results of all investigations of possible causes of heterogeneity among study results.  In a tinnitus neuroimaging study, authors present an explanation of results in terms of the heterogeneity of tinnitus, in terms of psychological and physical health, and laterality, loudness, and pitch of tinnitus.  “Our results demonstrate consistent disease-related effects of tinnitus across a heterogeneous population that varies in tinnitus sounds, loudness, laterality, and duration of tinnitus. Other medical and psychological comorbidities, such as head injury, hearing loss, depression, posttraumatic stress disorder, and anxiety, were not controlled in the current study; nor were the data acquisition techniques and data analytic approaches. Therefore, it is suggested that the identified regions from this meta-analysis, particularly from the cluster-level inference ALE with FWE, are invariant and shared across the spectrum of tinnitus patients. Findings are explained in the context of resting-state networks (RSNs), which are brain regions that exhibit paralleled activity while in a task-free state (Buckner et al., 2013; Husain & Schmidt, 2014). RSNs show typical spatial patterns and are identified by their associated sensory, motor, and cognitive functions (Smith et al., 2009).” | Discussion, Page 10 |
|  | 20d | Present results of all sensitivity analyses conducted to assess the robustness of the synthesized results.  N/A |  |
| Reporting biases | 21 | Present assessments of risk of bias due to missing results (arising from reporting biases) for each synthesis assessed.  In a tinnitus neuroimaging meta-analysis, authors utilized the fail-safe N method to insert noise and correct for biases. Results indicated that with an addition 6% noise, results remained consistent.  “The fail-safe N method assessed the robustness of the corrected ALE findings, which accounted for unpublished findings. A total of 6% noise was added to the meta-analysis, and results remained consistent regarding the significant cluster and five regions of convergence described above. However, when 11% of added noise was added to the meta-analytic data, these results were not replicated. “ | Results, Pages 8-9 |
| Certainty of evidence | 22 | Present assessments of certainty (or confidence) in the body of evidence for each outcome assessed.  Authors present an assessment of certainty by analyzing the data without correction for multiple comparisons, and results were replicated from the previous analyses.  “After implementation of an uncorrected ALE, results demonstrated 15 regions across 10 clusters. The first cluster contained the inferior parietal lobe and insula, while the second cluster replicated the findings from the corrected ALE. This particular cluster contained one region: the cingulate gyrus. Additional regions within the remaining nine clusters included the middle temporal gyrus, lingual gyrus, middle occipital gyrus, cuneus, medial frontal gyrus, subcallosal gyrus, and thalamus. Figure 3 shows the clusters that resulted from the single-threshold ALE. Coordinates and peak ALE scores from the single-threshold ALE can be seen in Table 3.” |  |
| **DISCUSSION** | | |  |
| Discussion | 23a | Provide a general interpretation of the results in the context of other evidence.  In a tinnitus neuroimaging meta-analysis, authors provide a general interpretation of the results.  “Our results demonstrate consistent disease-related effects of tinnitus across a heterogeneous population that varies in tinnitus sounds, loudness, laterality, and duration of tinnitus. Other medical and psychological comorbidities, such as head injury, hearing loss, depression, posttraumatic stress disorder, and anxiety, were not controlled in the current study; nor were the data acquisition techniques and data analytic approaches. Therefore, it is suggested that the identified regions from this meta-analysis, particularly from the cluster-level inference ALE with FWE, are invariant and shared across the spectrum of tinnitus patients. Findings are explained in the context of resting-state networks (RSNs), which are brain regions that exhibit paralleled activity while in a task-free state (Buckner et al., 2013; Husain & Schmidt, 2014). RSNs show typical spatial patterns and are identified by their associated sensory, motor, and cognitive functions (Smith et al., 2009).” | Discussion, Page 10 |
|  | 23b | Discuss any limitations of the evidence included in the review.  In a tinnitus neuroimaging meta-analysis, authors provide caveats to the results found.  “It is feasible that improvement of the spatial resolution of existing tools may lead to the identification of additional altered regions associated with tinnitus. Additionally, this meta-analysis did not control for demographic variables, psychological comorbidities, head injury, or differences in tinnitus percept or related distress. Future neuroimaging studies may account for these differences to map tinnitus in relation to specific comorbidities.” | Discussion, Page 16 |
|  | 23c | Discuss any limitations of the review processes used.  N/A |  |
|  | 23d | Discuss implications of the results for practice, policy, and future research.  Authors provide implications for future research.  “Future studies should aim to more fully characterize, structurally and functionally, altered brain regions indicative of disease-related effects of tinnitus, without the reliance on past assumptions related to the auditory network. These results provide compelling evidence that a paradigm shift is necessary in the field of tinnitus neuroimaging research. Investigators must recognize the effects from specific regions across canonical networks beyond the auditory resting-state network.  Functional modeling may help distinguish the relationship among the regions identified in this meta-analysis, including direct and indirect pathways involved in tinnitus generation, persistence, tolerance, bothersomeness, and habituation. Co-authors of this study aim to identify the functional pathways using functional meta-analytic connectivity modeling and structural equation modeling. By doing so, neuromodulatory therapies may become more refined and tailored to individuals’ needs regarding comorbid diagnoses and current health conditions.” | Discussion, Pages 15-16 |
| **OTHER INFORMATION** | | |  |
| Registration and protocol | 24a | Provide registration information for the review, including register name and registration number, or state that the review was not registered.  This meta-analysis was not registered. |  |
|  | 24b | Indicate where the review protocol can be accessed, or state that a protocol was not prepared.  A protocol was not prepared for this meta-analysis. |  |
|  | 24c | Describe and explain any amendments to information provided at registration or in the protocol.  N/A |  |
| Support | 25 | Describe sources of financial or non-financial support for the review, and the role of the funders or sponsors in the review.  Authors and funders report no financial support for the review.  “Funding sources had no role in the development of this manuscript. The content is solely the responsibility of the authors and does not necessarily represent the official views of the National Institutes of Health or the U.S. Government.” | Title Page, Page 1 |
| Competing interests | 26 | Declare any competing interests of review authors.  Authors report no competing interest.  “Authors report no conflicts of interest.” | Title Page, Page 1 |
| Availability of data, code and other materials | 27 | Report which of the following are publicly available and where they can be found: template data collection forms; data extracted from included studies; data used for all analyses; analytic code; any other materials used in the review.  Authors report that the data are publicly available and where this data can be found.  “The data that support the findings are available in Open Science Framework (Moring, 2022).” | Methods, Page 7 |

*From:*  Page MJ, McKenzie JE, Bossuyt PM, Boutron I, Hoffmann TC, Mulrow CD, et al. The PRISMA 2020 statement: an updated guideline for reporting systematic reviews. BMJ 2021;372:n71. doi: 10.1136/bmj.n71

For more information, visit: <http://www.prisma-statement.org/>
